# Influenza B Vaccines: Current Landscape and Novel Development Strategies

**DOI:** 10.3390/vaccines14070574

**Published:** 2026-06-29

**Authors:** Roman Y. Kotlyarov, Nikolai V. Ravin, Eugenia S. Mardanova

**Affiliations:** Institute of Bioengineering, Research Center of Biotechnology of the Russian Academy of Sciences, 119071 Moscow, Russia

**Keywords:** influenza, influenza B virus, vaccine, antigen

## Abstract

Influenza B virus (IBV) represents a significant global health threat, contributing 20–30% of annual influenza cases and causing substantial morbidity and mortality across all age groups. Current seasonal vaccines demonstrate variable effectiveness, highlighting the urgent need for next-generation approaches that provide enhanced and sustained protection against both IBV lineages. Moreover, continuous antigenic drift of circulating viruses progressively reduces the match between vaccine-induced antibodies and contemporary strains, necessitating broad-spectrum protection strategies. This review discusses influenza B virus control strategies, encompassing both conventional approaches and emerging vaccine technologies. While antiviral therapy, epidemiological surveillance, diagnostics, and non-pharmaceutical public-health measures are integral components of influenza B control, the present review focuses specifically on vaccine-based strategies. By critically appraising the available evidence, this review evaluates the extent to which these strategies may improve the effectiveness of IBV vaccines and, in the longer term, inform the prospect of reducing the burden of—or potentially eliminating—influenza B virus, a goal that remains hypothetical and requires clinical validation.

## 1. Introduction: Epidemiology and Public Health Burden of Influenza B Virus

### 1.1. Global Burden and Evolutionary Dynamics of Influenza Viruses

Influenza viruses pose a significant threat to global public health, causing annual seasonal epidemics of variable severity. These outbreaks result in approximately 3–5 million cases of severe respiratory illness and 290,000–650,000 deaths annually. Epidemics typically occur during winter months, when low temperatures and high humidity facilitate viral transmission [1,2].

The emergence of point mutations in surface glycoproteins, a phenomenon known as antigenic drift, occurs with relatively high frequency and leads to significant variability in morbidity and mortality rates across different years. In addition to seasonal epidemics, pandemics caused by novel influenza strains periodically emerge every few decades. In the 20th and early 21st centuries, pandemics were recorded in 1918, 1957, 1968, and 2009, resulting in millions of deaths worldwide [3]. The 1918 pandemic caused an estimated 50–100 million deaths globally, the 1957 pandemic resulted in approximately 1–2 million deaths, and the 1968 pandemic killed upwards of 1 million people [4,5,6]. Pandemic strains often arise through reassortment, or the exchange of genomic segments between two or more viral strains hosted by animals such as pigs or birds. This process, also referred to as antigenic shift, leads to substantial changes in the virus, rendering it distinct from its parental strains [7]. Consequently, populations lack significant immunity to these novel strains, resulting in increased morbidity, disease severity, and mortality [8,9].

Mass vaccination remains the most effective strategy for combating influenza outbreaks. Currently, the most widely used vaccines are inactivated formulations administered intramuscularly, although intranasal vaccines are also employed [10]. Vaccination induces immunity specific to the antigens included in the vaccine; however, continuous antigenic drift of circulating influenza viruses progressively reduces the match between vaccine-induced antibodies and contemporary strains, necessitating annual reformulation. In addition, vaccine-elicited immunity wanes over time, further diminishing protection. The World Health Organization (WHO) therefore issues biannual recommendations—in February for the Northern Hemisphere and in September for the Southern Hemisphere—specifying the strains to be included in the upcoming season’s vaccines. Given the variable efficacy of existing seasonal vaccines, significant efforts are underway to develop universal influenza vaccines capable of providing broad and long-lasting immunity [8,11].

Historically, pandemic preparedness and vaccine development efforts have been predominantly focused on influenza A viruses due to their zoonotic reservoir and pandemic potential. As a consequence, influenza B virus—despite its substantial contribution to seasonal morbidity and mortality—has received comparatively less attention as a target for next-generation vaccine strategies.

### 1.2. Epidemiological Significance of Influenza B Virus

Influenza B virus (IBV) is a prevalent respiratory pathogen that poses significant public health risks and contributes substantially to the morbidity and mortality observed during influenza epidemics. Studies indicate that 20–30% of all diagnosed influenza infections are attributable to IBV [12].

Influenza B virus was first identified in the 1940s and circulated as a single lineage until the mid-1970s to early 1980s, when two genetically and antigenically distinct lineages emerged: Victoria (named after B/Victoria/2/87) and Yamagata (named after B/Yamagata/16/88) [13]; the apparent recent extinction of the B/Yamagata lineage and its consequences for vaccine composition are addressed later in this section. The simultaneous circulation of these two phylogenetic lineages, which exhibit limited cross-reactivity and undergo independent antigenic drift, complicates the selection of vaccine strains and underscores the need for vaccines with broad-spectrum protection. This antigenic divergence persists in contemporary strains: the HA proteins of their current vaccine reference strains, B/Phuket/3073/2013 (B/Yamagata) and B/Austria/1359417/2021 (B/Victoria), share only ~92% amino-acid identity, with sequence variation concentrated in the immunodominant HA1 head domain and the HA2 stem domain highly conserved (Supplementary Figure S1). Furthermore, the regional variability in the prevalence of IBV strains can lead to mismatches between the WHO-recommended vaccine strains for an entire hemisphere and the viruses circulating in specific regions [14,15].

**Figure 1:**
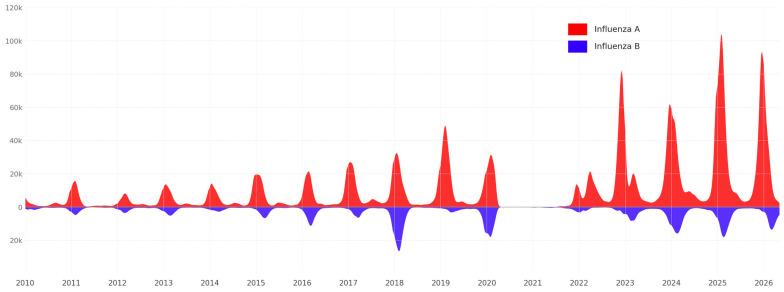
Statistics on Influenza A and Influenza B weekly cases of disease according to WHO data from 2010-01week to 2026-19week.

IBV typically co-circulates with IAV in the population, and during certain epidemics, IBV predominates [16,17]. This pattern is shaped in part by the comparatively lower transmissibility of influenza B: phylogeographically calibrated transmission models estimate a lower autumn–winter reproductive number for influenza B lineages than for influenza A subtypes [18]. Consistent with this, long-term surveillance analyses describe an inverse temporal relationship between the two types, with influenza B activity tending to rise during seasons or intervals in which influenza A circulation is low [19]. In certain years, the disease burden attributable to influenza B virus has been equivalent to that of influenza A virus (Figure 1). Influenza B was historically considered to cause less severe illness than influenza A; however, this assumption has been challenged by large population-based studies. An analysis of over 100,000 influenza-associated hospitalizations in the United States (2010–2019) found that patients hospitalized with influenza B had higher adjusted odds of ICU admission, mechanical ventilation, and death compared to those with A(H3N2), whereas A(H1N1)pdm09 was associated with the highest in-hospital severity among all types and subtypes [20]. Influenza B affects all age groups, with the highest incidence observed among children and young adults; it has been noted that B/Victoria tends to infect at a younger age than B/Yamagata. An analysis of US pediatric influenza-associated deaths from 2004 through 2012 demonstrated that influenza B was responsible for a substantial proportion of fatal cases in children, with the percentage varying by season [21]. In all but two seasons since national pediatric mortality surveillance began in the United States, influenza A viruses have been associated with more pediatric deaths than influenza B; the exceptions were the 2012–2013 and 2019–2020 seasons [22].

The 2017–2018 season saw IBV causing the highest number of severe cases. In European Union countries, 21% of patients in intensive care units with IBV infections succumbed to the disease, with 79% of IBV-related deaths occurring in patients over 60 years of age [23]. During the 2019–2020 influenza season, hospitalizations due to IBV were most frequent among individuals over 65 years old. Among children over 10 years of age, fatalities from IBV were more common than from IAV, and these children were more likely to require intensive care. Analysis of data from 2004 to 2019 revealed that 7–51% of influenza-related deaths in children were associated with IBV, except during the 2009–2010 pandemic [24]. According to WHO FluNet data, over the past 24 months, the contribution of IBV to influenza cases ranged from 1.7% (Week 50, 2025) to 68.2% (Week 19, 2026) [25].

A pivotal recent development in influenza B epidemiology has been the apparent extinction of the B/Yamagata lineage: no confirmed B/Yamagata isolates have been detected in global surveillance since March 2020, coinciding with the implementation of non-pharmaceutical interventions during the COVID-19 pandemic [26,27,28]. In response, the WHO recommended the exclusion of the B/Yamagata component from seasonal influenza vaccines beginning with the 2024–2025 Northern Hemisphere season, and regulatory agencies including the US FDA have endorsed the transition to trivalent formulations [29]. This event should be distinguished from the routine, drift-driven turnover of individual antigenic variants—whereby strains that have caused an epidemic are typically not re-detected in subsequent decades as population immunity accumulates [30]—since the loss of an entire lineage is of a qualitatively different order. Critically, and unlike influenza A, IBV is not sustained in an animal reservoir, so a strain that ceases to circulate in the human population has no zoonotic source from which to re-emerge; this feature renders the true elimination of a lineage, in principle, more attainable for influenza B than for any influenza A subtype. While the disappearance of an entire influenza lineage is unprecedented and raises the theoretical possibility of IBV elimination, the potential for re-emergence—whether from undetected circulation, laboratory stocks, or immune waning in unexposed birth cohorts—necessitates continued global surveillance and sustained investment in broadly protective IBV vaccine development [31,32]. The implications of B/Yamagata’s extinction for vaccine strategy, population immunity, and the evolutionary trajectory of the remaining B/Victoria lineage are discussed in the concluding sections of this review.

## 2. Strategies for Influenza B Virus Control

Control of influenza B virus relies on two complementary approaches: antiviral therapeutics and preventive mass immunization. The mainstay of antiviral therapy consists of neuraminidase inhibitors (NAIs)—oseltamivir (oral) and zanamivir (inhaled)—which suppress viral neuraminidase activity and prevent the release of progeny virions. These agents are used for both treatment and post-exposure prophylaxis, particularly in severely ill and high-risk patients. Notably, IBV neuraminidase exhibits reduced susceptibility to oseltamivir in vitro compared to influenza A NA, requiring higher drug concentrations for equivalent inhibition; zanamivir, by contrast, demonstrates more comparable activity against both types [33,34]. More recently, baloxavir marboxil—a first-in-class inhibitor of the cap-dependent endonuclease activity of the viral polymerase acidic (PA) protein—has been approved for the treatment of uncomplicated influenza and has demonstrated clinical efficacy against both influenza A and B viruses, including NAI-resistant strains [33]. A comprehensive review of antiviral therapeutics for influenza B is beyond the scope of this article; the following sections focus exclusively on vaccine-based strategies for IBV prevention and control.

### 2.1. Contemporary Seasonal Influenza Vaccines

**Figure 2:**
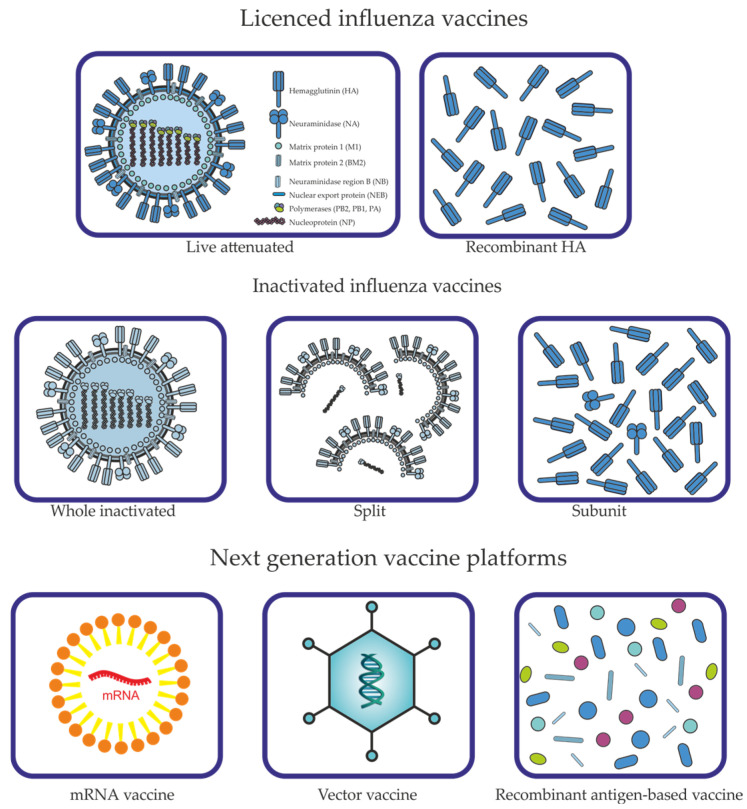
The influenza vaccine repertoire includes inactivated, live-attenuated, and recombinant protein vaccines, as well as next-generation candidates like mRNA vaccines, viral vector vaccines, and other antigen-based platforms.

Mass vaccination with seasonal influenza vaccines remains the primary strategy for controlling influenza B virus (IBV) infections. These vaccines are designed to confer protection against the most prevalent circulating strains. Currently, seasonal influenza vaccines that include coverage against IBV fall into three major categories: Inactivated Influenza Vaccines (IIV), Live Attenuated Influenza Vaccines (LAIV), and recombinant HA-based vaccines (rHA) [29,35,36,37] (Figure 2).

Until the 2023–2024 season, most licensed influenza vaccines were quadrivalent (QIV), containing antigens from two influenza A subtypes (A/H1N1pdm09 and A/H3N2) and both influenza B lineages (B/Victoria and B/Yamagata). Following the apparent extinction of the B/Yamagata lineage (discussed in Section 1.2), the WHO recommended the removal of the B/Yamagata component beginning with the 2024–2025 Northern Hemisphere season. The vaccines listed in Table 1 reflect the current and recently licensed product landscape; many of these formulations are in the process of transitioning from quadrivalent to trivalent composition.

The IIV group encompasses several subtypes:

Egg-based IIV: The virus is propagated in embryonated chicken eggs and chemically inactivated (e.g., with formaldehyde). These vaccines may be whole-virion, split-virus, or subunit formulations containing purified surface antigens—hemagglutinin (HA) and neuraminidase (NA). Examples include Fluarix Quadrivalent, Fluzone Quadrivalent, Flu-M tetra, Grippol, among others [35,38]. A recognized limitation of egg-based manufacturing is the acquisition of adaptive mutations during passage in embryonated eggs, which can alter the antigenicity of the vaccine virus relative to circulating strains. This liability arises upstream of conventional antigenic-drift considerations: a candidate vaccine virus must first be selected or adapted to replicate efficiently in embryonated eggs, and the mutations that confer egg-growth competence can simultaneously remodel antigenic surfaces, such that the propagated vaccine virus may already diverge antigenically from the original human isolate before the season’s drift is even taken into account [39]. While this phenomenon has been most extensively documented for influenza A(H3N2), a retrospective analysis of 16 influenza seasons (2002–2018) demonstrated that B/Victoria viruses are also susceptible to egg-adaptive antigenic changes: cell-propagated B/Victoria reference viruses were consistently more antigenically similar to circulating isolates than their egg-propagated counterparts [39]. This egg-adaptation liability provides an additional rationale for the development of cell-based and recombinant vaccine platforms, discussed below.

Cell-based IIV: The virus is cultured in mammalian cell lines (e.g., Madin–Darby Canine Kidney, MDCK), which reduces production time and eliminates the egg-adaptation mutations that can compromise antigenic fidelity. An example is Flucelvax Quadrivalent [40,41].

Recombinant HA: Produced by expressing the HA gene in insect cells (*Spodoptera frugiperda*, Sf9) using the baculovirus expression vector system (BEVS), followed by purification of the recombinant HA protein. These vaccines do not require live virus or eggs, making them suitable for individuals with egg allergies and eliminating the risk of egg-adaptation mutations. An example is Flublok Quadrivalent [36,42].

Adjuvanted IIV: Contain an immune-stimulating adjuvant (e.g., MF59 in Fluad Quadrivalent), enhancing the immune response. These vaccines are recommended for older adults to compensate for age-related immunosenescence [43,44].

The Live Attenuated Influenza Vaccine (LAIV) contains cold-adapted, temperature-sensitive attenuated viruses and is administered intranasally as a nasal spray. The IBV components of licensed LAIV formulations (e.g., FluMist Quadrivalent) are derived from the cold-adapted master donor virus B/Ann Arbor/1/66, whose internal gene segments confer the attenuated phenotype while the HA and NA genes were replaced with those of the recommended seasonal strains [45]. LAIV is approved for individuals aged 2 to 49 years but is not recommended for pregnant women or immunocompromised individuals. The intranasal route of administration enables the induction of mucosal immune responses, including secretory IgA, which may provide an additional layer of protection at the site of infection. Unlike parenterally administered inactivated vaccines, which elicit predominantly systemic IgG, intranasal vaccination induces local secretory IgA and tissue-resident memory responses at the respiratory mucosa—the portal of viral entry—and mucosal secretory IgA has been associated with cross-protective immunity against antigenically drifted strains [10,46]. The relative contribution of mucosal versus systemic immunity to protection against influenza B, and in particular to the interruption of transmission, remains incompletely defined and is an active area of investigation. This rationale has promoted the design of next-generation mucosal vaccine candidates discussed in Section 3. Different examples of currently used commercial vaccines are listed in Table 1.

### 2.2. Vaccine Effectiveness of Current Vaccines

The effectiveness of influenza B vaccines (vaccine effectiveness, VE) varies depending on the match between the vaccine strain and circulating virus, as well as the age and health status of the vaccinated population. Studies report that VE against influenza B ranges from 30% to 70%. For instance, in China, during the 2011–2012 season, VE among children aged 6–59 months was 67% (95% confidence interval [CI]: 41–82%) [47]. Similarly, in the United States, VE in children under 18 years of age during the 2019–2020 season reached 63% (95% CI: 38–78) [48]. In contrast, a study from Korea during the 2017–2018 season reported a VE of 29.7% (95% CI: −35.1 to 61.8) among children aged 6–59 months, suggesting a possible mismatch between vaccine and circulating strains [49].

A meta-analysis by Kalligeros et al. [50], covering the period from 2005 to 2019, estimated a pooled VE of 62.36% (95% CI: 54.50–70.22) against any influenza B–related hospitalization in fully vaccinated children. Among adults, VE also varied: a U.S.-based study estimated VE at 53% (95% CI: 37–64) during the 2019–2020 season among adults aged 18–64 years [51].

Quadrivalent vaccines offered broader protection by including strains from both B lineages, which was particularly important in seasons when both lineages co-circulated. However, strain mismatch—such as in the 2017–2018 season, when B/Yamagata predominated while the vaccine contained B/Victoria—can reduce vaccine effectiveness [49]. Nonetheless, even when the vaccine strain does not fully match the circulating virus, vaccination has been shown to reduce disease severity and the risk of complications such as hospitalization [52]. Direct head-to-head comparisons of vaccine effectiveness stratified simultaneously by vaccine platform and by influenza B lineage are, however, scarce in the published literature and constitute a recognized evidence gap. Quantitative effectiveness estimates for next-generation candidates are likewise not yet available, as the majority remain at the preclinical or early clinical stage, as indicated in the ‘Stage’ column of Table 2.

## 3. Modern Approaches to Vaccine Development

The limitations of current seasonal influenza vaccines—including suboptimal effectiveness, dependence on annual strain selection, and vulnerability to antigenic mismatch—have driven the development of next-generation vaccine strategies that target conserved viral antigens to provide broader and more durable protection. For influenza B virus, the rationale for such approaches is particularly compelling. Unlike influenza A, IBV does not maintain a sustained animal reservoir and does not undergo antigenic shift through reassortment with avian or swine viruses; consequently, IBV lacks pandemic potential but continues to impose a substantial burden through seasonal epidemics driven by antigenic drift within and between its two co-circulating lineages [13,53,54]. The relatively lower rate of antigenic drift and less extensive antigenic diversity of IBV compared to IAV suggest that the development of broadly protective—and potentially universal—influenza B vaccines may be a more tractable goal than for influenza A [54,55]. It should be emphasized, however, that this remains a biologically plausible hypothesis that has yet to be confirmed by clinical data and requires extensive clinical validation. Furthermore, the absence of a zoonotic reservoir raises the prospect that a sufficiently effective universal IBV vaccine could contribute not only to disease prevention but ultimately to the elimination of influenza B virus from the human population [53].

### 3.1. HA-Directed Vaccine Strategies

**Figure 3:**
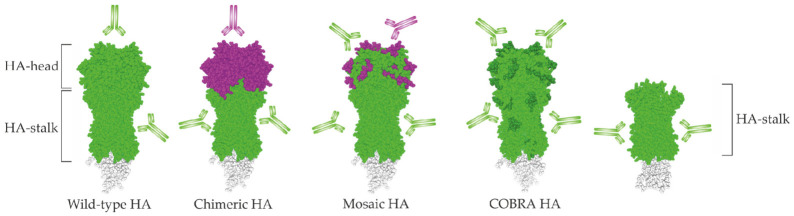
Strategies for focusing the immune response on conserved epitopes of HA. As an illustration, the 3D structure of HA from influenza B virus (B/Malaysia/2506/2004) was predicted using AlphaFold [57] and the model was visualized with SWISS-MODEL [58]. HA domains and antibody specificities are color-coded: green—wild-type HA and corresponding antibodies; magenta—modified HA and redirected antibodies; dark green—COBRA-modified HA antigen.

Hemagglutinin (HA) is the most abundant and immunodominant surface glycoprotein of the influenza virion. As the principal mediator of viral attachment and membrane fusion, HA is the primary target of neutralizing antibodies and the dominant immunogen in all currently licensed influenza vaccines. However, the globular head domain of HA—which harbors the major antigenic sites—undergoes continuous antigenic drift, necessitating annual vaccine reformulation and contributing to suboptimal vaccine effectiveness. To overcome these limitations, several innovative strategies have been developed that aim to redirect immune responses toward more conserved HA epitopes or to engineer broadly reactive HA antigens capable of eliciting cross-lineage protection against both B/Victoria and B/Yamagata influenza B viruses [8,56] (Figure 3).

#### 3.1.1. Chimeric Hemagglutinins (cHAs)

Chimeric hemagglutinins (cHAs) are genetically engineered proteins in which the globular head domain of hemagglutinin (HA) from one influenza strain is replaced with that of another, while the stem (stalk) domain is retained. Since the HA head harbors the major strain-specific antigenic sites and is subject to continuous antigenic drift, whereas the stem domain is structurally and antigenically more conserved, sequential immunization with cHAs sharing the same stem but presenting different heads redirects the immune response toward the conserved stem, thereby eliciting broadly cross-reactive antibodies. The underlying mechanism relies on the principle of immune subdominance: upon each successive immunization, the head-specific B cells elicited by the previous construct fail to recognize the new, heterologous head domain and are not boosted, whereas B cells targeting the shared stem epitopes are restimulated at each round, progressively accumulating and dominating the response. This approach has been validated for influenza A group 1 viruses through phase I–II clinical trials, which demonstrated that adjuvanted cHA vaccines induce durable, broadly protective anti-stalk antibody responses in humans [59,60].

For influenza B virus, the cHA strategy exploits the conservation of the IBV HA stem domain between the B/Victoria and B/Yamagata lineages. In this context, cHA constructs are engineered by grafting exotic influenza A head domains (to which the human population is immunologically naive) onto the influenza B HA stem. Ermler et al. [61] provided proof-of-concept for this approach by constructing a panel of influenza B cHAs bearing head domains from various influenza A subtypes. Sequential vaccination of mice with these constructs induced broadly reactive antibodies directed against the conserved IBV stem domain. Critically, these stem-specific antibodies conferred protection against lethal challenge with viruses from both the Victoria and Yamagata lineages, demonstrating cross-lineage protective efficacy within a single immunization regimen [61].

Despite these encouraging preclinical results, several important gaps remain. All clinical evaluations of the cHA platform to date have employed influenza A group 1 constructs (H1 stalk); cHA vaccines based on the influenza B HA stem have not yet advanced to clinical testing. Furthermore, compared to the extensively characterized influenza A HA stem, the repertoire of broadly neutralizing antibodies targeting the IBV stem remains notably limited (see Section 3.1.4), and the immunological consequences of pre-existing IBV immunity on stalk-directed responses in humans are poorly understood [53,54]. Recent advances in combining the cHA approach with mucosal delivery using attenuated ΔNS1 viral backbones—which induce robust CD8^+^ T-cell responses in addition to humoral immunity and have shown cross-protection against both influenza A and B viruses in animal models—suggest that intranasal cHA-based platforms may be particularly suitable for extending this strategy to influenza B [62]. The clinical translation of influenza B–specific cHA vaccines therefore represents a promising yet underexplored avenue for universal IBV vaccine development.

#### 3.1.2. Mosaic Hemagglutinins (mHAs)

The term “mosaic” in the context of influenza vaccine design has been applied to describe distinct but related strategies. In its original and most widely used sense—and as discussed in this section—a mosaic hemagglutinin (mHA) is a single engineered HA molecule in which the major strain-specific antigenic sites have been replaced with heterologous sequences (typically from exotic influenza A HAs), while the conserved framework and stem regions are retained. This intramolecular redesign redirects the immune response away from the hypervariable head epitopes and toward the more conserved HA regions shared across diverse influenza B strains.

The proof-of-concept for intramolecular mosaic HA vaccines against influenza B was established by Sun et al. [63], who engineered mHA constructs by replacing four principal antigenic regions of IBV HA with corresponding sequences from influenza A HAs (H5, H8, H11, H13). The resulting mosaic HAs were expressed as soluble trimers and used to sequentially immunize mice, eliciting high titers of cross-reactive antibodies. These antibodies mediated antibody-dependent cellular cytotoxicity (ADCC) in vitro and conferred protection against both homologous and heterologous influenza B challenges, including viruses of both lineages. Importantly, this platform was proposed as a foundation for a trivalent universal vaccine covering both influenza A and B strains [63].

González-Domínguez et al. [64] subsequently refined this approach, developing mosaic HA-based inactivated influenza B vaccines in both whole-virion inactivated (WIV) and split formulations. Mosaic HAs were engineered by replacing the major antigenic sites of IBV HA with sequences from influenza A HAs (H8, H13, H5). A critical advance of this study was the systematic evaluation of adjuvant effects: formulation with a TLR9 agonist (CpG 1018) promoted enhanced Th1 responses and improved in vivo cross-protection, whereas an MF59-like oil-in-water nano-emulsion (AddaVax) broadened humoral immunity. Both adjuvanted formulations elicited significantly higher antibody titers against conserved epitopes, including the stem, compared to conventional vaccines, and conferred cross-protection against antigenically diverse strains spanning B/Lee/1940 to contemporary isolates. Importantly, these vaccines were also evaluated in pre-immune mice—a model designed to recapitulate human pre-existing IBV immunity—with assessments of both innate and cellular immune contributions [64].

More recently, the mosaic antigen strategy has been extended to a mucosal live attenuated influenza vaccine (LAIV) platform. Han et al. [63] employed an iterative genetic algorithm to design mosaic HA and NA sequences derived from B/Victoria lineage isolates (2009–2021), with the explicit objective of maximizing T-cell epitope coverage rather than solely redirecting the humoral response toward conserved B-cell epitopes. The resulting mosaic LAIV candidate (MoBV) was administered intranasally to mice and compared with conventional inactivated and live attenuated vaccines. MoBV induced higher cross-reactive antibody responses (HAI, microneutralization, and NAI titers), markedly elevated mucosal IgA that persisted for over 100 days, and enhanced T-cell responses including tissue-resident memory populations. Upon lethal challenge with seven antigenically diverse IBV strains, MoBV conferred substantially broader protection than conventional LAIV or IIV: the high-dose MoBV group (10^7^ EID_50_) achieved 80–100% survival against six of seven challenge strains, whereas LAIV protected against only three and IIV against two. This study represents the first application of the mosaic approach to an intranasal LAIV format specifically for influenza B and underscores the potential of mucosal delivery to induce both systemic and local immunity—a critical advantage for respiratory pathogens [65].

The mosaic HA concept has also been implemented on a virus-like particle (VLP) scaffold. Zhao et al. [66] constructed VLPs incorporating two mosaic HA proteins designed to present a broader range of epitopes from both B/Victoria and B/Yamagata lineages. In murine immunization studies, the mosaic VLPs induced balanced Th1/Th2 cytokine responses and conferred effective protection against homologous IBV challenge, with notably reduced lung pathology. Of particular interest, the B/Victoria-derived mosaic component exhibited significant cross-reactivity against B/Yamagata strains, outperforming a commercial quadrivalent inactivated vaccine (QIV) in eliciting broad humoral responses—further underscoring the capacity of the mosaic approach to achieve cross-lineage immunity [66].

A conceptually distinct but complementary strategy was described by Gu et al. [13], who applied targeted residue substitutions to generate hybrid HAs that merge the antigenic features of both Victoria and Yamagata lineages within a single HA molecule. Unlike the classical mosaic approach—which replaces antigenic sites with exotic influenza A sequences to redirect immunity toward conserved framework regions—the hybrid HA strategy aims to create a novel antigenic surface that simultaneously engages lineage-specific antibody responses against both B/Victoria and B/Yamagata. The investigators compared amino acid differences in the HA proteins of B/Phuket/3073/2013 (Yamagata) and B/Washington/02/2019 (Victoria) and identified key residues for targeted replacement. From numerous candidate designs, two hybrid-HA constructs were selected and evaluated in a ferret model, where they conferred superior cross-lineage protection against heterologous challenge compared to wild-type-based vaccines, including against circulating strains with divergent HA sequences. This approach holds particular promise for rapid adaptation within cell-culture and recombinant vaccine production platforms in response to future influenza B antigenic evolution [13].

Collectively, these studies demonstrate that the mosaic HA strategy—originally conceived as a single proof-of-concept—has now been validated across multiple vaccine formats (recombinant trimers, inactivated WIV/split, VLPs, and live attenuated vaccines), delivery routes (intramuscular and intranasal), and adjuvant systems (CpG 1018, AddaVax). The extension to mucosal LAIV platforms capable of inducing secretory IgA and tissue-resident memory is particularly noteworthy, as it addresses a recognized limitation of parenteral vaccination for respiratory pathogens. When considered alongside the complementary hybrid HA approach, the mosaic strategy provides a versatile toolkit for engineering broadly protective influenza B immunogens that can be adapted to diverse production and delivery platforms.

#### 3.1.3. Computationally Optimized Broadly Reactive Antigen (COBRA) Hemagglutinins

The Computationally Optimized Broadly Reactive Antigen (COBRA) methodology represents a distinct bioinformatics-driven approach to universal influenza vaccine design. Unlike chimeric or mosaic HA strategies that redirect immune responses toward conserved epitopes by replacing or masking immunodominant domains, the COBRA approach generates entirely novel HA sequences through iterative rounds of multiple consensus sequence alignments derived from phylogenetically diverse influenza isolates. The resulting synthetic HA proteins incorporate the most prevalent amino acid residues at each position, effectively representing an “averaged” antigenic surface that retains key functional properties—including receptor binding and fusogenic activity—while broadening the spectrum of elicited antibodies. Originally developed and validated for H5N1 avian influenza using virus-like particle (VLP) platforms [67], the COBRA methodology has since been successfully applied to H1N1, H3N2, and H2 subtypes of influenza A virus, consistently demonstrating superior breadth of hemagglutination inhibition (HAI) antibody responses compared to wild-type HA antigens [68,69,70].

The application of COBRA to influenza B virus was first reported by Carlock and Ross [71], who designed three novel IBV HA immunogens derived from both B/Victoria and B/Yamagata lineage sequences. These COBRA HA proteins were expressed on VLPs and evaluated in ferrets pre-immunized with historical B/Victoria or B/Yamagata viruses—a model that recapitulates the immunological imprinting observed in the human population. Critically, ferrets vaccinated with B-COBRA HA VLPs generated neutralizing antibodies with high HAI titers against a broad panel of influenza B viruses from both lineages, irrespective of their pre-immune history. In contrast, VLPs expressing wild-type IBV HA preferentially boosted antibody responses against viruses of the same lineage, with little or no seroprotective titers against the mismatched lineage. These findings demonstrated that a single COBRA-designed IBV HA antigen can overcome the lineage-specific bias imposed by prior exposure, a significant advantage over conventional vaccine approaches [71].

More recently, Carlock et al. [72] further investigated the interplay between COBRA B-HA immunogens and pre-existing immunity in a murine model. This study compared the recall B-cell responses elicited by B-COBRA-2 (BC2), a broadly reactive IBV HA antigen, with those induced by modern wild-type HA vaccines from each lineage in mice with varying immunological backgrounds to B/Victoria, B/Yamagata, or both. The broadly reactive BC2 antigen stimulated significantly higher levels of IgM-secreting antibody-secreting cells (ASCs) compared to wild-type HA antigens, suggesting enhanced de novo B-cell activation rather than mere recall of lineage-restricted memory. This property is relevant in the context of annual influenza vaccination, where pre-existing immunity can constrain the breadth of the response to updated antigens through several distinct processes. Immune imprinting (historically termed “original antigenic sin”) refers specifically to the durable, biasing influence of the first antigenic encounters in infancy and early childhood, which preferentially shape responses to antigenically related strains encountered later in life [73,74]. Superimposed on this, repeated exposures in later life progressively remodel—and frequently focus—the polyclonal B-cell repertoire through additional mechanisms, including competition among B-cell clones of differing affinity, preferential recall and expansion of pre-existing memory, antigenic seniority, and epitope masking by circulating antibodies (antibody feedback), rather than through imprinting per se [73,75]. The net effect of immune history on response breadth is therefore context-dependent and may either enhance or limit reactivity to drifted or mismatched antigens [75,76]. By presenting an optimized antigenic consensus surface, the COBRA approach may help to overcome these constraints and restore broad cross-lineage reactivity against influenza B virus [72].

To date, COBRA-designed IBV HA immunogens have been evaluated exclusively in preclinical models (mice and ferrets); clinical trials have not yet been initiated, representing a critical next step for this platform.

#### 3.1.4. Headless HA and Stem-Focused Vaccine Strategies (HA Stem-Based Immunogens)

An alternative vaccine design strategy focuses exclusively on the conserved stem (stalk) region of hemagglutinin (HA), omitting the highly variable globular head domain. By removing the immunodominant head, this approach aims to focus immune responses on the conserved stem region, which harbors epitopes less prone to antigenic drift, thereby eliciting broadly cross-reactive antibodies. The foundational rationale for this strategy in the context of influenza B virus was provided by Dreyfus and colleagues, who identified a panel of human monoclonal antibodies targeting conserved HA epitopes. Among these, CR9114—a broadly neutralizing antibody that recognizes an epitope in the HA stem—was shown to bind and neutralize influenza viruses across both IAV groups 1 and 2 as well as IBV. Critically, passive transfer of CR9114 protected mice from lethal challenge with viruses of both the B/Victoria and B/Yamagata lineages, providing the first direct evidence that stem-directed antibodies alone can confer cross-lineage protection against influenza B [77].

Building on this concept, Zeng et al. [78] developed a rationally designed stem immunogen specifically for IBV. Utilizing structure predictions generated by AlphaFold2, the authors engineered B60-Stem-8071—a headless HA stem construct derived from B/Brisbane/60/2006, stabilized by grafting a peptide segment corresponding to the CR8071 antibody epitope as a structural linker. A key achievement of this work was the soluble expression of B60-Stem-8071 as a trimeric protein in E. coli, demonstrating feasibility for cost-effective and scalable production—a significant practical advantage over eukaryotic expression systems. In murine immunization studies, B60-Stem-8071 elicited cross-reactive antibodies and conferred protection against lethal challenge with representative strains of both the Victoria and Yamagata lineages. This study provided proof-of-concept for the application of computational structure prediction tools to the rational design of IBV stem-based vaccine immunogens [78].

Despite these encouraging findings, the stem-based approach for influenza B remains at an early stage of development compared to analogous strategies for influenza A. Several limitations and research gaps warrant consideration. First, although additional stalk-binding neutralizing antibodies against the IBV HA have been described—notably 5A7, which targets a conserved epitope near the C-terminus of HA1 [79], and the TRL845/TRL848/TRL849 series isolated by single-cell screening [80]—CR9114 remains the most extensively characterized stem-directed antibody relevant to IBV: its conserved pan-influenza stem epitope and fusion-inhibiting mechanism have been defined at atomic resolution by X-ray crystallography (obtained with influenza A hemagglutinins, with influenza B engagement demonstrated by electron microscopy), whereas the epitopes of 5A7 and the TRL antibodies have been localized only by mutational or peptide-based mapping [77]. The repertoire of stem epitopes structurally defined on the IBV HA therefore remains remarkably narrow. This contrasts sharply with the situation for influenza A group 1 viruses, where a diverse and extensively characterized panel of IGHV1-69-biased stem-directed bnAbs has been described [81]. This limited epitope landscape raises the question of whether the IBV stem presents a sufficient breadth of neutralizing targets for robust vaccine-elicited protection. Reliance on a single conserved epitope class is, moreover, intrinsically escape-prone: because protection would rest on a narrow antibody specificity, a single amino-acid substitution within the targeted stem epitope could in principle abrogate binding—an argument for combining stem-directed immunogens with additional conserved targets (e.g., the NA active site and M2e) in multivalent or multi-epitope formats, as empirically supported by formulations in which the combination of HA stem with M2e raised survival against IBV challenge to 90% (Section 3.5) [82]. Second, the structural stabilization of the HA stem domain in the absence of the head remains technically challenging; the head domain contributes significantly to the conformational integrity of the HA trimer, and its removal can expose non-native surfaces that may elicit non-neutralizing or poorly protective antibody responses. The AlphaFold2-guided engineering strategy employed by Zeng et al. represents a promising solution, but further optimization and validation of stem conformation fidelity are required [78]. Third, all available data for IBV stem-based immunogens derive exclusively from murine models; evaluation in ferrets—the preferred preclinical model for influenza transmission and pathogenesis—and ultimately in clinical trials has not yet been undertaken. By comparison, stem-focused nanoparticle immunogens for influenza A have progressed to evaluation in non-human primates [83] and are approaching clinical development. The translation of these more advanced platforms—including ferritin-based and computationally designed two-component nanoparticles—to the influenza B HA stem represents an obvious yet unexplored research direction. Bridging this gap will be essential for determining whether stem-based approaches can achieve clinically meaningful cross-lineage protection against IBV.

### 3.2. Vaccines Based on Neuraminidase (NA)

Neuraminidase (NA) has long been a “forgotten antigen” in influenza vaccinology. Despite evidence dating back over 50 years that anti-NA immunity independently contributes to protection against influenza, the NA content of licensed inactivated and live attenuated vaccines is neither standardized nor routinely quantified—in contrast to hemagglutinin (HA), for which potency is defined by single radial immunodiffusion [56,84]. This historical neglect reflects both the immunodominance of HA in the context of current vaccine formulations and the lack, until recently, of scalable assays for measuring NA-specific antibody responses. As a result, anti-NA immunity elicited by conventional seasonal vaccines is inconsistent and often insufficient [85]. For influenza B virus, the NA-directed approach is particularly attractive: the enzymatic active site and surrounding epitopes of IBV NA exhibit substantially higher conservation between the B/Victoria and B/Yamagata lineages than do the corresponding antigenic sites of the HA head domain, and broadly cross-reactive monoclonal antibodies targeting these conserved NA regions have been identified [54,86,87]. These findings, together with accumulating preclinical evidence that NA-specific immunity can reduce disease severity and viral shedding even in the absence of matched HA antibodies, have motivated the development of vaccine strategies that incorporate NA as a primary—rather than incidental—immunogen.

#### Conserved NA Epitopes as Targets for Cross-Lineage Protection Against Influenza B

The rationale for targeting IBV neuraminidase rests on accumulating evidence—primarily from monoclonal antibody (mAb) studies—that conserved NA epitopes can elicit cross-lineage immunity. Although NA-directed antibodies are generally not considered capable of preventing viral attachment and entry, immunity to NA has been shown to reduce disease severity, lower viral titers in the lungs, and decrease viral shedding in animal models [54,56]. This section reviews the key evidence defining the epitope landscape of IBV NA and assesses the current—still early—stage of translating these findings into vaccine candidates.

The foundational evidence for broadly protective IBV NA epitopes was provided by Wohlbold et al. [86], who generated a panel of five murine monoclonal antibodies against influenza B NA. These antibodies bound to and inhibited NA from viruses of both the Victoria and Yamagata lineages, conferring in vivo protection in mice against strains spanning over 70 years of IBV evolution (1940s–2010s). Electron microscopy mapping revealed that their targets were conserved epitopes on the NA head distinct from the catalytic site [86]. In a complementary study, Madsen et al. [87] isolated seven human monoclonal antibodies following natural influenza B infection. Two of them, 1G05 and 2E01, inhibited NA enzymatic activity across strains from both lineages, from the prototypic 1940 B/Lee isolate to contemporary circulating viruses. Sequence analysis confirmed that the key epitope residues targeted by these antibodies remain highly conserved among current IBV strains, supporting the concept that conserved NA sequences are sufficiently similar between the two lineages to serve as viable vaccine targets [87].

More recently, structural and immunological studies have substantially expanded our understanding of the molecular basis for broad NA reactivity. Madsen et al. [88] demonstrated that even conventional egg-based seasonal vaccines can—albeit rarely—elicit broadly protective NA antibodies: mAb-297, isolated from plasmablasts of vaccinated individuals, inhibited NA activity across both influenza A (including H5N1) and influenza B viruses by targeting a conserved motif within the NA active site. This finding underscores both the feasibility and the current inefficiency of inducing such responses with existing vaccine formulations [88]. Independently, Jo et al. [89] resolved cryo-electron microscopy structures of the human antibody DA03E17 in complex with NA from A/H1N1, A/H3N2, and B/Victoria-lineage viruses, revealing a recurring Asp-Arg (DR) motif in CDR H3 that mimics sialic acid interactions with catalytic residues. Notably, B-cell receptor sequences containing this motif were identified across all donors in a healthy human repertoire database, suggesting that such broadly reactive precursors may be relatively common and amenable to vaccine-mediated expansion [89]. In parallel, Lederhofer et al. [90] characterized a panel of human monoclonal antibodies that target the conserved catalytic site of neuraminidase (NCS mAbs); these antibodies recognized NA from multiple influenza A subtypes as well as influenza B, and conferred prophylactic protection in mice against H1N1, H5N1, and IBV challenge. Cryo-EM structural analysis revealed that these antibodies achieve broad inhibition through water-mediated substrate mimicry at the conserved catalytic pocket—a mechanism with direct implications for rational immunogen design [90].

These structural insights are complemented by functional evidence of cross-lineage NA-directed immunity at the polyclonal level. Page et al. [91] demonstrated that immunity induced by Victoria-lineage viruses generated NA-specific antibodies conferring strong cross-protection against Yamagata-lineage challenge in animal models. In contrast, Yamagata-induced responses provided substantially weaker cross-lineage protection. The authors attributed this asymmetric cross-protection to differences in NA-specific antibody breadth: Victoria-elicited antibodies exhibited higher reactivity to Yamagata NA than vice versa. This observation has potential implications for vaccine strain selection and may contribute to the understanding of competitive dynamics between the two IBV lineages [91].

Despite the compelling epitope-level rationale established by these studies, the translation of IBV NA into dedicated vaccine candidates remains at a notably early stage compared to HA-directed strategies. To date, no standalone IBV NA vaccine immunogen has been advanced to preclinical or clinical evaluation. Rather, IBV NA has been incorporated as one component within broader multi-antigen formulations. Pardi et al. [92] included IBV NA alongside HA, NP, and M2 in a pentavalent nucleoside-modified mRNA-LNP vaccine, and Le et al. [93] demonstrated that mRNA encoding IBV NA fused to the M2 ectodomain, when combined with conventional inactivated vaccine, enhanced both NA- and HA-specific antibody responses and improved cross-lineage protection in mice (see Section 3.3). In a recent preclinical study, Catani et al. [94] evaluated octavalent vaccine formulations—both adjuvanted recombinant protein and mRNA-LNP formats—containing HA and NA from all four seasonal influenza strains, including influenza B. Both formats conferred complete protection against homologous IBV challenge, with the mRNA-LNP formulation eliciting higher serum IgG titers against both HA and NA. However, in all of these studies, the independent contribution of the IBV NA component to protection was not delineated from that of co-administered HA or other antigens.

This contrasts with the more advanced state of NA-directed vaccine development for influenza A, where the COBRA methodology (described in Section 3.1.3 for HA) has been applied to generate broadly cross-reactive N1 and N2 neuraminidase antigens. COBRA N1 NA elicited NAI antibodies against a broad panel of HxN1 strains and protected mice and ferrets from both seasonal H1N1 and pre-pandemic H5N1 challenge [95,96], while COBRA N2 NA induced cross-reactive antibodies against antigenically diverse H3N2 viruses and reduced lung viral titers in mice [97]. When these COBRA NA antigens were integrated alongside COBRA HA proteins—including a COBRA IBV HA component—into multivalent intranasal formulations, the resulting vaccines conferred protection against influenza B virus challenge in ferrets [98,99]. However, it is important to note that in all such formulations, influenza B is represented exclusively by a COBRA-designed HA immunogen, with no COBRA-derived IBV neuraminidase component; protection against IBV challenge in these studies is therefore attributable to anti-HA rather than anti-NA immunity. A COBRA neuraminidase antigen specific to influenza B virus has not yet been reported, nor have standalone recombinant IBV NA immunogens—whether displayed on nanoparticle scaffolds, VLPs, or as stabilized soluble tetramers—been systematically evaluated for cross-lineage protective efficacy. Given the high conservation of IBV NA epitopes documented by monoclonal antibody studies and the emerging structural understanding of the molecular determinants of broad NA inhibition, the development of dedicated IBV NA vaccine immunogens represents a logical and timely priority for the field.

### 3.3. mRNA Vaccines

The advantages of the mRNA platform for influenza vaccine development—including rapid design and manufacturing timelines, avoidance of egg-adaptation mutations, and the capacity to encode multiple antigens within a single formulation—have been extensively reviewed [100] and are now being translated to clinical development against influenza B virus. However, clinical evaluation of mRNA-based seasonal influenza vaccines has revealed a consistent and yet incompletely understood challenge: the IBV hemagglutinin components elicit weaker immune responses compared to their influenza A counterparts. This discrepancy—referred to here as the “IBV immunogenicity gap”—has emerged as a central obstacle for the platform and is a recurring theme across multiple clinical programs.

#### 3.3.1. Clinical-Stage Seasonal mRNA Influenza Vaccines

The most advanced clinical data for mRNA-based seasonal influenza vaccines targeting IBV are available for two quadrivalent candidates: mRNA-1010 (Moderna) and the nucleoside-modified mRNA (modRNA) vaccine developed by Pfizer/BioNTech.

mRNA-1010 encodes the hemagglutinin of four seasonal influenza strains (A/H1N1, A/H3N2, B/Victoria, and B/Yamagata) delivered via lipid nanoparticles (LNP). Phase I/II trials (NCT04956575) demonstrated an acceptable safety profile and robust immunogenicity; however, seroconversion rates and geometric mean titers (GMT) for influenza B strains did not consistently surpass those of the licensed comparator vaccine [101,102]. Two subsequent Phase III trials (NCT05415462 and NCT05566639, enrolling >14,000 adults) confirmed this pattern: mRNA-1010 elicited immune responses that were statistically noninferior and superior to both standard-dose and high-dose licensed vaccines for influenza A strains, yet responses against influenza B strains remained lower relative to the active comparator [103]. In contrast, a separate Phase III safety and immunogenicity trial (P303; NCT05827978) evaluating an optimized mRNA-1010 formulation demonstrated statistically noninferior and superior HAI responses against all four vaccine-matched strains—including both influenza B components—compared to both standard-dose and high-dose licensed comparators, suggesting that formulation optimization may address the IBV immunogenicity gap observed with the original candidate [104]. An analysis of hemagglutination inhibition (HAI) titers as correlates of protection demonstrated that this metric retains its predictive value for mRNA-1010, similar to licensed vaccines [103]. Most recently, a pivotal Phase III efficacy trial (P304; NCT06602024) enrolling more than 40,000 adults aged ≥50 years across 11 countries demonstrated a relative vaccine efficacy (rVE) of 26.6% (95% CI: 16.7–35.4%) compared to a standard-dose licensed comparator, with consistent efficacy across individual strains including B/Victoria (rVE 29.1%) [105]. These data were presented at the 10th ESWI Influenza Conference and IDWeek 2025; a peer-reviewed publication of the full efficacy analysis is anticipated.

The Pfizer/BioNTech modRNA quadrivalent influenza vaccine (qIRV) has followed a parallel development trajectory. A Phase I/II dose-finding study (NCT05052697) in adults aged 18–85 years evaluated multiple dose levels (30 µg and 60 µg) against a licensed high-dose comparator (Fluzone HD). The qIRV induced higher HAI titers and fold-rises for influenza A strains compared to the comparator; however, no consistent immunogenic advantage was observed for the B/Victoria or B/Yamagata components. Notably, cell-mediated immune responses—including CD4^+^ and CD8^+^ T-cell activation—were higher against all four strains, suggesting that HAI titers may not fully capture the immune benefits of mRNA vaccination against IBV [106]. The subsequent Phase III trial (NCT05540522), published in the New England Journal of Medicine [107], enrolled 18,476 adults aged 18–64 years. The modRNA vaccine demonstrated statistically superior relative efficacy compared to a licensed inactivated vaccine (rVE 34.5%; 95% CI: 7.4–53.9%), driven predominantly by protection against A/H3N2 and A/H1N1 strains, with almost no influenza B cases detected during the 2022–2023 season. Critically, noninferiority of HAI antibody responses was demonstrated for influenza A strains but not for the B strains [107]. These findings mirror those of mRNA-1010 and underscore the cross-platform nature of the IBV immunogenicity gap.

#### 3.3.2. The IBV Immunogenicity Gap: Possible Mechanisms

The observation that mRNA-encoded IBV HA consistently underperforms IAV HA in eliciting HAI antibody responses has been noted across both the Moderna and Pfizer programs as well as in systematic reviews [103,108]. Several non-mutually exclusive hypotheses may account for this phenomenon. First, intrinsic differences in the glycosylation patterns and conformational dynamics of IBV HA expressed from mRNA—relative to IAV HA—could affect antigen presentation or epitope accessibility. Second, immune imprinting from prior IBV exposure may shape the recall response to mRNA-encoded HA in ways that differ from the response to IAV, where imprinting patterns are better characterized. Third, the current reliance on HAI titers as the primary immunogenicity endpoint may inadequately capture the full spectrum of mRNA vaccine-elicited immunity against IBV, particularly cell-mediated and non-neutralizing antibody responses [106,107]. Resolution of this question is critical for the rational design of next-generation mRNA influenza vaccines.

#### 3.3.3. Next-Generation mRNA Formulations Addressing IBV Immunogenicity

Several strategies are being pursued to overcome the IBV immunogenicity gap. Moderna has evaluated next-generation seasonal mRNA formulations incorporating modified strain compositions. In a Phase I/II trial (NCT05827068), pentavalent (mRNA-1011.1, adding an additional A/H3N2 strain) and quadrivalent or pentavalent formulations replacing B/Yamagata with additional A/H3N2 strains (mRNA-1011.2, mRNA-1012) were assessed in adults aged 50–75 years. Removal of the B/Yamagata component did not diminish the antibody response to B/Victoria, providing immunological support for the regulatory transition to trivalent formulations following B/Yamagata’s apparent extinction [109]. Additionally, the combination mRNA-1083 vaccine—encoding hemagglutinins of four influenza strains alongside SARS-CoV-2 spike protein—demonstrated noninferiority and, for three of four influenza strains including B/Victoria, superiority of immune responses compared to separate administration of licensed influenza and COVID-19 vaccines in a Phase III trial enrolling over 8000 adults aged ≥50 years [110].

A distinct antigen engineering approach to the IBV immunogenicity problem has been described by Thornhill-Wadolowski et al. [111], who designed fusion proteins in which multiple HA ectodomains are linked by T4 fibritin foldon trimerization domains, enabling expression of multimerized HA antigens from a single mRNA species. In preclinical studies, this next-generation seasonal influenza vaccine elicited HAI titers significantly higher than those induced by Fluzone High-Dose across all seasonal strains, including the B/Victoria component—a notable improvement over the consistently lower B-strain responses observed with first-generation mRNA vaccines [111]. If validated clinically, this antigen multimerization strategy may represent a generalizable solution to the IBV immunogenicity gap.

#### 3.3.4. Multi-Antigen mRNA Approaches for IBV

The mRNA platform also enables the co-encoding of multiple IBV antigens beyond hemagglutinin, an approach that may broaden the protective immune response. Pardi et al. [92] described a pentavalent nucleoside-modified mRNA-LNP vaccine incorporating HA from both IBV lineages together with neuraminidase (NA), nucleoprotein (NP), and matrix protein 2 (M2), demonstrating proof-of-concept for multi-antigen IBV mRNA vaccination (see Section 3.2 and Section 3.5 for additional discussion). Le et al. [93] further showed that mRNA encoding IBV NA fused to the M2 ectodomain, when administered alongside conventional inactivated vaccine, enhanced both NA- and HA-specific antibody responses and improved cross-lineage protection in mice (see Section 3.2) [93]. These combinatorial approaches suggest that the integration of NA-encoding mRNA into seasonal vaccine formulations may partially compensate for suboptimal HA-directed responses and broaden the spectrum of anti-IBV immunity.

### 3.4. Vector-Based Vaccines

Viral vector platforms offer distinct advantages for influenza B vaccine development that complement the antigen-focused strategies described in preceding sections. By delivering genetic cargo encoding influenza antigens within the context of a viral backbone, these platforms elicit robust CD8^+^ T-cell responses in addition to humoral immunity—a property that is particularly relevant for targeting conserved internal proteins such as nucleoprotein (NP), which are poorly immunogenic when administered as recombinant proteins [112,113]. Moreover, many vector-based vaccines are amenable to intranasal administration, enabling the induction of mucosal IgA and tissue-resident memory T cells at the site of respiratory infection [112]. For influenza B virus, where the limited antigenic diversity of internal proteins such as NP may permit cross-lineage T-cell recognition with a single construct, these attributes make vector-based approaches an appealing strategy.

The most extensively characterized vector-based IBV vaccines employ adenoviral platforms to express nucleoprotein. Kim et al. [114] demonstrated that a single intranasal dose of a recombinant human adenovirus serotype 5 (Ad5) vector carrying the IBV NP gene elicited strong NP-specific humoral and CD8^+^ T-cell responses in mice. Notably, complete protection against both B/Victoria and B/Yamagata lineage viruses was achieved exclusively via the intranasal route, whereas intramuscular administration was less effective—underscoring the importance of mucosal delivery for respiratory pathogens. Lee et al. [115] further showed that Ad5 vectors encoding NP from either the Victoria or Yamagata lineage induced CD8^+^ T cells capable of cross-recognizing NP epitopes from both lineages, confirming that the degree of NP conservation between the two IBV lineages is sufficient for cross-lineage T-cell–mediated protection. To circumvent the limitation of pre-existing anti-Ad5 immunity in the human population—a major obstacle for human adenoviral vectors—Wang et al. [116] engineered a bovine adenovirus (type 3) vector expressing NP genes from both influenza A and influenza B fused to an autophagy-inducing C5 peptide. A single intranasal dose of this bivalent formulation conferred complete protection against a broad spectrum of influenza A and B viruses in mice and a two-dose intranasal regimen cross-protected ferrets against seasonal strains [116].

More recently, vector-based approaches for IBV have expanded beyond NP-only designs to incorporate surface glycoproteins. Niu et al. [117] constructed a tetravalent vaccine, AdC-Flu-Tet, based on a single chimpanzee adenoviral vector (AdC68) encoding the hemagglutinins of both B/Yamagata and B/Victoria lineages alongside H1N1 NP in the ΔE1 region, with M2e epitopes from H1N1 and H3N2 displayed on the fiber protein. This multi-antigen single-vector design induced both humoral and cellular immune responses and conferred full protection against H1N1, H3N2, and both IBV lineages in a murine model [117]. Wang et al. [118] subsequently developed Ad-Hex, a hexavalent vaccine comprising three chimpanzee adenoviral vectors, each encoding two HA genes: Ad-H1H3, Ad-BYBV (carrying both IBV lineage HAs), and Ad-H5H7. A single intranasal dose provided complete protection against all six vaccine-matched strains and 60% survival against mismatched strains in mice; mechanistic analyses demonstrated that the vaccine activated germinal center B-cell responses against both cognate HAs and the conserved HA stalk, driving production of cross-reactive antibodies [118]. These studies demonstrate that chimpanzee adenoviral vectors—which circumvent the pre-existing anti-human Ad5 immunity—can accommodate complex multi-antigen payloads sufficient for simultaneous protection against both IBV lineages as well as influenza A subtypes.

The Modified Vaccinia Ankara (MVA) vector has also been explored as a platform for influenza vaccines incorporating IBV-derived antigens. Mintaev et al. [119] developed rMVA-k1-k2, a universal epitope-based vaccine in which computationally selected B-cell and T-cell epitopes from conserved regions of NP, M1, and HA from both influenza A and B viruses were expressed from an MVA backbone. Double immunization protected mice against influenza A viral pneumonia with ≥67% efficiency, with protection levels comparable to those of the M-001 and MVA-NP+M1 vaccines; however, direct challenge with influenza B viruses was not reported [119]. The most clinically advanced MVA-based influenza vaccine, MVA-NP+M1 (developed by the Jenner Institute/Vaccitech), encodes NP and M1 derived from influenza A (A/Panama/2007/99, H3N2). In the INVICTUS Phase II trial, MVA-NP+M1 was co-administered with standard seasonal vaccine to adults aged ≥65 years; however, the trial was stopped after a single season because the recommended annual influenza vaccine was changed, and safety of the new combination had not been established [120]. Importantly, the IBV cross-protective capacity of MVA-NP+M1 has not been directly evaluated, as neither the immunogens nor the challenge endpoints were IBV-specific; moreover, the NP and M1 sequences encoded by MVA-NP+M1 (derived from A/Panama/2007/99, H3N2) share only approximately 20% amino acid identity with their influenza B virus counterparts, suggesting that meaningful T-cell cross-reactivity between IAV and IBV through this construct is unlikely [120]. Thus, while MVA remains a promising vector platform, its application to influenza B virus remains largely unexplored.

Despite the encouraging preclinical data reviewed above, several limitations constrain the clinical translation of vector-based IBV vaccines. First, pre-existing immunity to human adenoviral vectors—particularly Ad5, which has been widely used in gene therapy and vaccine trials—remains a significant concern, as it can attenuate transgene-specific immune responses [112,113]; the use of chimpanzee and bovine adenoviral vectors represents a practical solution, but these platforms require independent clinical safety evaluation. Second, with the exception of the underpowered MVA-NP+M1 trial, no vector-based vaccine specifically targeting influenza B has advanced to clinical testing. Third, while NP-directed T-cell immunity clearly contributes to cross-lineage protection in murine models, evidence from multiepitope vaccine trials (discussed in Section 3.5) suggests that T-cell immunity alone may be insufficient for clinically meaningful protection and is most effective when combined with antibody-inducing components. The recent emergence of multi-antigen vector designs—such as AdC-Flu-Tet and Ad-Hex—that co-deliver HA alongside conserved internal proteins may address this limitation by simultaneously engaging both humoral and cellular arms of the immune response.

### 3.5. Recombinant Protein Vaccines

A variety of expression systems can be employed for vaccine production, including mammalian cell cultures, insect cell cultures, yeast, bacteria, and plants. Each system offers distinct advantages in terms of safety, post-translational modifications, scalability, and production costs, allowing for flexibility in vaccine design and manufacturing. Several expression systems are employed for influenza B vaccine production. Full-length influenza B virus proteins as well as truncated fragments served as antigens. An example of a recombinant vaccine incorporating influenza B virus antigens is the licensed quadrivalent vaccine Flublok. It contains full-length hemagglutinins from four influenza strains, two of which are derived from influenza B virus (B/Victoria and B/Yamagata). The vaccine is produced in *Spodoptera frugiperda* insect cell culture.

Considerable efforts are focused on the use of conserved influenza B virus antigens for the development of broadly protective vaccines against various influenza virus strains. Conserved antigens are generally short in length and, as a result, exhibit low immunogenicity. Different strategies are utilized to improve the effectiveness of these vaccines. One such approach is the incorporation of multiple epitopes designed to elicit both T-cell and B-cell immune responses.

The Multimeric-001 (M-001) vaccine is designed as a universal influenza vaccine candidate. It comprises nine conserved B- and T-cell epitopes derived from the HA, M1, and NP proteins of both influenza A and B viruses, and is produced in E. coli [121]. However, despite its capacity to elicit broad humoral and cellular immune responses, this immunogenicity was insufficient to confer statistically significant protection against clinical disease in a large Phase III trial [122].

FLU-v is a synthetic universal influenza vaccine candidate containing four conserved epitopes derived from NP, M1, and M2 proteins of influenza A and B viruses to induce T-cell immunity. Adjuvanted FLU-v has been shown to elicit enhanced IFN-γ and Th1 cellular responses in a Phase IIb trial. However, the trial was not powered for efficacy, and in an exploratory efficacy analysis these immunogenicity findings did not translate into statistically significant protection against clinical disease [123].

Another candidate, a multi-epitope vaccine (MEV) containing 37 immunodominant B- and T-cell epitopes from influenza A and B viruses HA and NA (including 19 B-cell, 7 CD4+ T-cell, and 11 CD8+ T-cell epitopes, nine of which are from influenza B virus) [124], was generated in E. coli. Immunized C57BL/6 mice challenged with 10 LD50 of H1N1, H3N2, and both influenza B lineages (Victoria and Yamagata) exhibited 100% survival and robust T-cell responses [125].

In several studies, influenza B virus proteins were used to generate virus-like particles (VLPs). Unlike monomeric recombinant proteins, VLPs offer the advantage of presenting viral epitopes in a native-like conformation, which effectively triggers both B-cell and T-cell responses. VLPs have demonstrated their effectiveness as a platform for antigen presentation against various infections [126].

VLPs formed by consensus N1 NA (cN1), N2 NA (cN2), and influenza B NA (B cNA), alongside tandem repeat 5×M2e and M1, were produced in insect cells. Mice vaccinated with m-cNA-M2e VLP were protected against a broad spectrum of influenza A viruses (including H1N1, H3N2, H5N1, H7N9, and H9N2 subtypes) as well as both Yamagata and Victoria lineages of influenza B virus [127].

Using self-assembling norovirus P protein VLP platform, consensus sequences from the conserved stalk HA2 domain of influenza A/H1N1, A/H3N2, and influenza B viruses were displayed. Immunization of mice with these chimeric particles elicited robust, subtype-specific IgG responses. Furthermore, following viral challenge, vaccinated mice exhibited significantly reduced pulmonary viral titers [53].

Virus-like nanoparticles based on two self-assembling bacterial proteins—E2p from Bacillus stearothermophilus and I3-01 from Thermotoga maritima—were used to display M2e peptides from both the influenza B virus and the influenza A virus. In mice, this single-component vaccine, produced in CHO cells, conferred protection against challenge with 10 × LD50 of mouse-adapted A/Puerto Rico/8/1934 (H1N1) or 5 × LD50 of mouse-adapted B/Florida/4/2006 (Yamagata lineage) [128].

A self-assembling recombinant human heavy chain ferritin cage was used to display three highly conserved influenza epitopes: the A α-helix of hemagglutinin, M2e, and a conserved neuraminidase region. Chimeric nanoparticles were produced in E. coli. Despite containing no influenza B virus-specific epitopes, immunization with chimeric nanoparticles protected mice against lethal challenge with both influenza A and B viruses [129].

In another study, the de novo design of protein nanoparticles for multivalent antigen presentation was carried out. The focus was on presenting antigens in trimeric conformations. Two-component nanoparticles were developed, consisting of a trimeric component and a pentameric component [130]. The trimeric component was produced in mammalian cell culture, while the pentameric component was produced in E. coli.

The authors genetically fused HA ectodomains from the four strains included in the licensed 2017–2018 seasonal influenza vaccine to the N-terminus of the trimeric component. Following in vitro co-assembly of the trimeric and pentameric components, the vaccine candidate was evaluated in multiple animal models, including mice, ferrets (Mustela putorius), and non-human primates (NHPs; *Macaca mulatta*). Various influenza virus strains were used in the study, such as H1N1, H3N2, H5N1, H7N9, B/Yamagata, and B/Victoria [131]. The vaccine elicited antibody responses against vaccine-matched viruses and protected animals from lethal viral challenge. However, despite the broad range of viruses tested, protective efficacy against influenza B viruses was not demonstrated, and this issue requires further investigation.

A nanoparticle-based vaccine platform, created via solid-phase peptide synthesis (SPPS), was designed to present both linear and helical epitopes. Using this platform, broadly protective influenza vaccines were developed. Immunization with conserved epitopes (HA stem, NA active site, and M2e) resulted in 50–75% survival against influenza B and H1N1 challenge. Notably, combining HA stem and M2e antigens improved protection, achieving 90% survival [82].

The outer membrane protein complex (OMPC) of Neisseria meningitidis was used as a carrier to enhance the immunogenicity of a conserved HA cleavage-site peptide. This peptide, which is also a component of the M-001 vaccine [132] and an MVA-based vaccine candidate [119], was produced by standard solid-phase synthesis and coupled to OMPC. The resulting conjugate was highly immunogenic and provided 100% protection against lethal influenza B virus infection in vivo (BALB/c mice) [133].

**Table 2:** Pipeline of IBV-relevant vaccine candidates discussed in this review: clinical-stage and advanced preclinical programs.

Candidate/Platform	Antigen(s)	IBV Antigen(s)	Stage	Model	Key IBV-Relevant Finding	Reference
**HA-Directed Strategies**						
IBV cHA panel *chimeric HA; recombinant protein/virus*	IBV HA stem + exotic IAV head domains (H5, H7, H8)	HA stem (B/Vic + B/Yam conserved)	**Preclinical**	Mice	Sequential immunization with cHAs bearing different IAV heads on IBV stem induced broadly reactive stem-directed antibodies; cross-lineage protection against both B/Victoria and B/Yamagata lethal challenge.	[61]
Mosaic HA trimers *recombinant soluble trimers*	IBV mHA: antigenic sites replaced with IAV HA (H5, H8, H11, H13)	IBV HA framework + stem	**Preclinical**	Mice	Cross-reactive Abs with ADCC activity; protection against both IBV lineages. Proposed as foundation for trivalent universal IAV+IBV vaccine.	[63]
Mosaic HA—WIV/split *inactivated vaccine*	IBV mHA (H8, H13, H5 head replacements) ± adjuvants	IBV HA conserved regions	**Preclinical**	Mice (naïve + pre-immune)	Cross-protection spanning B/Lee/1940 to contemporary isolates; CpG 1018 enhanced Th1 responses; AddaVax broadened humoral immunity. Evaluated in pre-immune model.	[64]
MoBV *mosaic LAIV; intranasal*	Mosaic HA + NA (B/Vic-derived, genetic algorithm–designed)	Mosaic HA + NA (B/Victoria)	**Preclinical**	Mice	First mosaic LAIV for IBV. Mucosal IgA persisting >100 days; enhanced HAI, NAI, T_RM_ responses; 25–100% survival against multiple IBV strains via intranasal route.	[65]
Mosaic HA VLPs *virus-like particles*	Two mosaic HA proteins (B/Vic + B/Yam epitopes) on VLP	Mosaic HA (dual-lineage)	**Preclinical**	Mice	Balanced Th1/Th2 responses; B/Vic mosaic component showed significant cross-reactivity to B/Yam; outperformed commercial QIV in breadth of humoral response.	[66]
Hybrid HA *targeted residue substitution*	Hybrid HA merging B/Vic + B/Yam antigenic features	Hybrid HA (B/Phuket + B/Washington)	**Preclinical**	Ferrets	Superior cross-lineage protection against heterologous challenge vs. wild-type HA vaccines; compatible with cell-culture and recombinant production platforms.	[13]
B-COBRA HA *COBRA consensus; VLP*	Computationally optimized IBV HA consensus sequences on VLPs	COBRA IBV HA (B/Vic + B/Yam derived)	**Preclinical**	Ferrets (pre-immune); mice	Overcame the lineage-specific bias imposed by prior exposure; broad cross-lineage HAI titers irrespective of prior exposure history; enhanced de novo B-cell activation (IgM ASCs) vs. wild-type HA.	[71,72]
B60-Stem-8071 *headless HA stem; recombinant*	IBV HA stem (headless), AlphaFold2-designed, trimeric	HA stem (B/Brisbane/60/2006)	**Preclinical**	Mice	Cross-lineage protection against B/Vic + B/Yam challenge; soluble trimeric expression in *E. coli*—proof-of-concept for computational stem immunogen design.	[78]
**mRNA Vaccines**						
mRNA-1010 *Moderna; LNP*	HA × 4 (H1, H3, B/Vic, B/Yam)	HA (B/Vic + B/Yam)	**Phase III**	Adults ≥50 yrs (N = 40,000)	Pivotal efficacy trial (P304): rVE 26.6% (95% CI: 16.7–35.4%) vs. licensed SD comparator; B/Vic rVE 29.1%. Original formulation (P301/P302) showed an “IBV immunogenicity gap”—HAI responses to B strains consistently lower than to A strains vs. comparator; the optimized formulation (P303) demonstrated superior HAI responses for all four strains including both B lineages.	[101,103,104,105]
modRNA qIRV *Pfizer/BioNTech; LNP*	HA × 4 (H1, H3, B/Vic, B/Yam)	HA (B/Vic + B/Yam)	**Phase III**	Adults 18–64 yrs (N = 18,476)	rVE 34.5% (95% CI: 7.4–53.9%) vs. licensed IIV; driven by A/H3N2+H1N1. HAI noninferiority NOT met for B strains; enhanced CD4^+^/CD8^+^ T-cell responses against all strains.	[106,107]
mRNA-1011/mRNA-1012 *Moderna next-gen; LNP*	HA × 4–5 (modified compositions ± B/Yam)	HA (B/Vic)	**Phase I/II**	Adults 50–75 yrs	Removal of B/Yam component did not diminish B/Vic antibody response—immunological support for transition to trivalent formulations.	[109]
mRNA-1083 *Moderna combo flu + COVID; LNP*	HA × 4 + SARS-CoV-2 spike	HA (B/Vic + B/Yam)	**Phase III**	Adults ≥50 yrs (N > 8000)	Noninferior/superior immune responses for 3/4 flu strains including B/Vic vs. separate administration of licensed flu + COVID vaccines.	[110]
Pentavalent IBV mRNA *mRNA-LNP*	HA (B/Vic + B/Yam), NA, NP, M2	HA (B/Vic + B/Yam), NA, NP, M2	**Preclinical**	Mice	Multi-antigen IBV mRNA proof-of-concept; co-encoding of 5 IBV antigens in a single LNP formulation.	[92]
IBV NA-M2e mRNA *mRNA-LNP + IIV*	IBV NA fused to M2 ectodomain (mRNA) + conventional IIV	NA (IBV) + M2e (IAV)	**Preclinical**	Mice	NA-M2e mRNA supplementation enhanced both NA- and HA-specific Ab responses and improved cross-lineage IBV protection when combined with IIV.	[93]
Multimerized HA mRNA *T4 fibritin foldon; mRNA*	Multiple HA ectodomains linked by T4 fibritin trimerization domains	HA (B/Vic component)	**Preclinical**	Mice	HAI titers significantly higher than Fluzone HD for all strains including B/Vic—potential solution to the IBV immunogenicity gap.	[111]
Octavalent HA+NA *protein + mRNA-LNP formats*	HA + NA × 4 seasonal strains (AF03-adjuvanted protein and mRNA-LNP)	HA + NA (IBV)	**Preclinical**	Mice	Complete protection vs. homologous IBV challenge; mRNA-LNP format elicited higher IgG vs. adjuvanted protein for both HA and NA.	[94]
**Viral Vector-Based Vaccines**						
Ad5-IBV NP *human Ad5; intranasal*	IBV NP	NP	**Preclinical**	Mice	Complete cross-lineage protection against B/Vic + B/Yam exclusively via intranasal route; cross-lineage CD8^+^ T-cell recognition of NP epitopes from both lineages.	[114,115]
BAd-C5-NP/A + BAd-C5-NP/B *bovine Ad3; intranasal*	NP (IAV + IBV) + C5 autophagy peptide	NP	**Preclinical**	Mice; ferrets	Single intranasal dose in mice: complete protection against broad-spectrum IAV + IBV; prime-boost (2 doses) in ferrets: cross-protection against seasonal strains. Bypasses pre-existing anti-human Ad5 immunity.	[116]
AdC-Flu-Tet *chimpanzee AdC68; single vector*	HA (B/Yam + B/Vic) + H1N1 NP + M2e (H1N1, H3N2)	HA (B/Vic + B/Yam)	**Preclinical**	Mice	Full protection against both IBV lineages + H1N1 + H3N2 from a single chimpanzee adenoviral vector encoding multiple antigens.	[117]
Ad-Hex *3× chimpanzee Ad vectors; intranasal*	HA × 6: Ad-H1H3, Ad-BYBV (B/Yam + B/Vic), Ad-H5H7	HA (B/Vic + B/Yam)	**Preclinical**	Mice	Single intranasal dose: 100% protection vs. all 6 matched strains; 60% vs. mismatched; GC B-cell activation and cross-reactive stalk Abs.	[118]
rMVA-k1-k2 *MVA vector*	Computed B-cell + T-cell epitopes from NP, M1, HA (IAV + IBV)	Epitopes from IBV NP, M1, HA	**Preclinical**	Mice	≥67% protection vs. IAV pneumonia. *IBV challenge not performed.*	[119]
MVA-NP+M1 *MVA; Vaccitech*	NP + M1 (IAV H3N2-derived only)	None (IAV-derived)	**Phase II ^a^**	Adults ≥65 yrs	Trial stopped after 1 season; underpowered. *IBV cross-protection not evaluated*—immunogens and endpoints were IAV-specific.	[120]
**Recombinant Protein/Peptide/Nanoparticle Vaccines**						
M-001 (Multimeric-001) *recombinant protein; E. coli*	9 conserved B-cell + T-cell epitopes from HA, M1, NP (IAV + IBV)	Epitopes from IBV HA, M1, NP	**Phase III ^b^**	Adults	Broad humoral + cellular immunogenicity but *no statistically significant clinical protection* in Phase III.	[121,122]
FLU-v *synthetic peptide*	4 conserved epitopes from NP, M1, M2 (IAV + IBV)	Epitopes from IBV NP, M2	**Phase IIb**	Adults	Enhanced IFN-γ; reduced viral shedding on H3N2 challenge. 0/58 IBV cases in non-adjuvanted group; no statistical significance for clinical protection.	[123]
MEV *multi-epitope; recombinant; E. coli*	37 epitopes from HA + NA (IAV + IBV): 19 B-cell, 7 CD4^+^, 11 CD8^+^	9 epitopes from IBV	**Preclinical**	Mice	100% survival against H1N1, H3N2, B/Victoria, and B/Yamagata (10 LD_50_ challenge); robust T-cell responses.	[124,125]
m-cNA-M2e VLP *VLP; insect cells*	Consensus N1 + N2 + B NA; 5×M2e tandem repeat; M1	Consensus IBV NA	**Preclinical**	Mice (young + aged)	Broad protection against IAV (H1N1, H3N2, H5N1, H7N9, H9N2) + both IBV lineages; comparable efficacy in aged mice.	[127]
E2p/I3-01 M2e-BM2 nanoparticle *self-assembling; CHO cells*	M2e (IAV) + BM2 ectodomain (IBV)	BM2 ectodomain	**Preclinical**	Mice	Protection against IAV (H1N1, 10×LD_50_) + IBV Yamagata lineage (5×LD_50_) challenge.	[128]
OMPC–HA cleavage peptide *peptide conjugate*	Conserved HA cleavage-site peptide conjugated to *N. meningitidis* OMPC	HA0 cleavage-site peptide (conserved)	**Preclinical**	Mice	100% protection against lethal IBV challenge.	[133]
SPPS epitope nanoparticle *solid-phase peptide synthesis*	HA stem + NA active site + M2e epitopes	HA stem + NA active site	**Preclinical**	Mice	50–75% survival vs. IBV challenge; HA stem + M2e combination achieved 90% survival. Novel HA stem epitope conferred 100% survival.	[82]
Ferritin HA/M2e/NA nanoparticle *ferritin cage; E. coli*	HA α-helix + M2e + conserved NA region (IAV-derived)	None specifically ^c^	**Preclinical**	Mice	Cross-protection against lethal IAV + IBV challenge despite no IBV-specific epitopes—suggests shared conserved determinants.	[129]
Quadrivalent HA nanoparticle *de novo two-component; VRC/UW*	HA ectodomains × 4 seasonal strains on designed nanoparticle	HA (B/Vic + B/Yam)	**Preclinical**	Mice; ferrets; NHPs	Potent Ab responses + stem-directed cross-reactive Abs against IAV. *Protective efficacy against IBV not demonstrated.*	[131]

**Stage: Preclinical Phase I/II Phase II Phase IIb Phase III Phase III (failed). **^a^ this trial was stopped after a single season because the recommended annual influenza vaccine was changed, and safety of the new combination had not been established. ^b^ M-001 Phase III trial (NCT03450915) did not demonstrate statistically significant clinical protection despite robust immunogenicity. ^c^ Contains only IAV-derived epitopes; IBV cross-protection attributed to shared conserved determinants between IAV and IBV. **Note on NA-directed vaccines:** No standalone IBV neuraminidase vaccine immunogen has been advanced to preclinical or clinical evaluation to date. IBV NA appears as a co-antigen in multi-antigen formulations listed under their respective platform categories [92,93,94,127]. See Section 3.2 for a detailed discussion of conserved IBV NA epitope characterization and the rationale for dedicated NA-directed vaccine development. **Abbreviations:** Ab, antibody; ADCC, antibody-dependent cellular cytotoxicity; Ad, adenovirus; ASC, antibody-secreting cell; BM2, influenza B M2 protein; COBRA, Computationally Optimized Broadly Reactive Antigen; cHA, chimeric hemagglutinin; GC, germinal center; HA, hemagglutinin; HAI, hemagglutination inhibition; IBV, influenza B virus; IAV, influenza A virus; IIV, inactivated influenza vaccine; LAIV, live attenuated influenza vaccine; LNP, lipid nanoparticle; M2e, matrix protein 2 ectodomain; mHA, mosaic hemagglutinin; MVA, Modified Vaccinia Ankara; NA, neuraminidase; NAI, neuraminidase inhibition; NHP, non-human primate; NP, nucleoprotein; OMPC, outer membrane protein complex; QIV, quadrivalent inactivated vaccine; rVE, relative vaccine efficacy; T_RM_, tissue-resident memory T cells; VLP, virus-like particle; VRC, Vaccine Research Center; UW, University of Washington; WIV, whole-virion inactivated; SPPS, solid-phase peptide synthesis; CHO, Chinese hamster ovary; modRNA, nucleoside-modified messenger RNA; qIRV, quadrivalent influenza modRNA vaccine.

## 4. Future Prospects

The development of effective vaccines against influenza B virus remains a multifaceted challenge that reflects both the unique biology of IBV and the broader limitations of current influenza vaccination strategies.

*Limitations of the evidence base*. Several limitations of the evidence synthesized in this review should be acknowledged. Much of the epidemiological data on influenza B burden and lineage circulation derives from passive surveillance systems that are subject to underreporting, geographically uneven testing infrastructure, evolving diagnostic practices, and the substantial disruption to global influenza surveillance caused by the COVID-19 pandemic [25,26].

*The B/Yamagata extinction and its implications*. The apparent disappearance of B/Yamagata lineage viruses from global circulation since March 2020 represents an unprecedented event in influenza epidemiology [27,28,31]. This has prompted regulatory action: the WHO recommended the exclusion of B/Yamagata from seasonal vaccines beginning with the 2024–2025 Northern Hemisphere season, and the US ACIP (Advisory Committee on Immunization Practices) endorsed the transition to trivalent formulations for the 2025–2026 season [29]. Notably, serological studies demonstrate that population-level antibody titers against B/Yamagata HA and NA remain elevated despite several years of non-circulation [134], a finding that may reflect cross-reactive boosting by B/Victoria exposure or the longevity of immunological memory. The immunological asymmetry between the two lineages—wherein Victoria-induced NA-specific antibodies confer superior cross-lineage protection compared to Yamagata-induced responses—has been proposed as a contributing factor to Yamagata’s competitive displacement [45,91]. Nevertheless, the possibility of B/Yamagata re-emergence from laboratory stocks or undetected reservoirs necessitates continued surveillance and preparedness [31,32].

*Rebalancing the antigenic focus*. A recurring theme across multiple vaccine platforms reviewed herein is the critical importance of anti-NA immunity. Conventional inactivated and live attenuated vaccines fail to elicit robust NA-specific antibody responses, whereas recombinant NA-based formulations provide superior cross-protection against antigenically divergent IBV strains in preclinical models. Monoclonal antibody studies have identified conserved NA epitopes—including but not limited to the catalytic site—that are preserved across both lineages spanning over 70 years of viral evolution [86,87]. The integration of NA as a co-equal immunogen alongside HA—whether through recombinant protein supplementation, mRNA co-encoding, or nanoparticle co-display—emerges as a key strategic priority for next-generation IBV vaccines [85,93].

*Conserved epitope-based and multiepitope approaches.*

Substantial progress has been achieved in identifying and validating conserved IBV epitopes suitable for universal vaccine design. Multiepitope vaccine platforms—including M-001, FLU-v, MVA-based constructs, and the MEV formulation—have shown that combining B-cell, CD4+, and CD8+ T-cell epitopes from both surface and internal proteins can elicit broad humoral and cellular immunity [119,121,124,125]. However, clinical translation has proven challenging: the M-001 Phase III trial (NCT03450915) failed to demonstrate statistically significant clinical protection despite robust immunogenicity, highlighting the gap between immune correlates measured in trials and real-world protective efficacy [122]. The FLU-v platform demonstrated reduction in viral shedding and symptom severity in human challenge studies, though its efficacy specifically against IBV requires further validation [135,136]. These results suggest that T-cell–mediated immunity, while contributing to disease amelioration, may be insufficient as a standalone protective mechanism and is most effective when combined with antibody-inducing components.

*Nanoparticle and VLP platforms*. Self-assembling nanoparticles and virus-like particles offer versatile scaffolds for multivalent display of conserved influenza epitopes. Ferritin-based nanoparticles presenting HA stem, M2e, and NA epitopes, as well as VLPs co-displaying M2e tandem repeats with consensus neuraminidase sequences, have demonstrated broad cross-protection against both IAV and IBV in murine models [127,129]. Chemically synthesized epitope nanoparticles targeting combinations of HA stem, NA active site, and M2e epitopes achieved up to 100% survival against lethal IBV challenge in specific formulations [82]. These platforms are particularly attractive due to their modularity, scalability, and capacity to present antigens in a repetitive array that enhances B-cell activation.

*Computational approaches to immunogen design*. Bioinformatics and structural prediction tools are increasingly shaping the design of next-generation IBV vaccine immunogens. Structure prediction platforms, particularly AlphaFold2, have enabled the rational engineering of stabilized stem-based immunogens that retain native conformation while achieving soluble expression in prokaryotic systems—a critical advantage for cost-effective, large-scale production [78]. In parallel, the Computationally Optimized Broadly Reactive Antigen (COBRA) methodology—which generates consensus HA sequences through iterative phylogenetic alignment—has yielded IBV HA immunogens capable of overcoming the lineage-specific bias imposed by prior exposure and eliciting broad cross-lineage neutralizing antibody responses in pre-immune animal models [71,72]. Together, these computational approaches—structure-guided stabilization and consensus-based antigen optimization—provide complementary tools for designing immunogens with both improved breadth and manufacturability across diverse delivery platforms.

*mRNA technology: promise and current limitations*. The mRNA platform offers undeniable advantages for influenza vaccine development, including rapid design and manufacturing timelines, avoidance of egg-adaptation mutations, and the capacity to encode multiple antigens simultaneously [100]. Clinical evaluation of mRNA-1010 (Moderna) and the Pfizer/BioNTech modRNA vaccine has confirmed acceptable safety profiles and immunogenicity against influenza A strains. However, both candidates have consistently demonstrated reduced immunogenicity against IBV components compared to IAV in Phase III trials [103,107,108]. Whether this reflects intrinsic antigenic properties of IBV HA, immune imprinting effects, or limitations of current HAI-based correlates of protection remains to be elucidated. Multi-antigen mRNA formulations incorporating NA, NP, and M2 alongside HA represent a promising direction for broadening the immune response beyond HA alone [92,93].

*Toward an integrated vaccine strategy*. The evidence reviewed herein suggests that no single antigenic target or platform is likely to achieve universal, durable protection against IBV in isolation. Rather, the most promising path forward involves the integration of complementary approaches: (i) HA-directed immunity broadened through chimeric, mosaic, or computationally designed immunogens targeting conserved epitopes across both lineages; (ii) NA-specific responses elevated to co-equal status with HA immunity through recombinant supplementation or multi-antigen encoding; (iii) T-cell immunity against conserved internal proteins (NP, M1) providing an additional layer of heterosubtypic protection; and (iv) optimized delivery systems—including mRNA-LNP, nanoparticles, VLPs, and mucosal (intranasal) platforms—selected to maximize both systemic and mucosal immune responses. Beyond antigen design, the attenuated influenza backbone itself can serve as a mucosal vector for multivalent combination vaccines: a modified trivalent LAIV encoding conserved SARS-CoV-2 T-cell epitopes within its A/H1N1 and A/H3N2 components—while retaining an unmodified B/Victoria strain—conferred combined protection against seasonal influenza, including influenza B, and COVID-19 in a preclinical (Syrian hamster) model [137]. Although the IBV component was not itself engineered in that work, the same vectoring strategy is in principle extensible to the B lineage, offering a route to broaden or prolong B-directed immunity within a single mucosal platform—a prospect that remains to be realized. Crucially, an integrated strategy does not imply that all platforms are equally validated: the approaches reviewed here differ markedly in evidentiary maturity—from mRNA formulations, which have generated large randomized Phase III efficacy data (albeit with a consistent IBV immunogenicity gap; Section 3.3), to chimeric, stem-based, and nanoparticle immunogens that remain at the preclinical stage. Prioritization within an integrated framework should therefore be weighted by the maturity, reproducibility, and risk profile of the supporting clinical evidence. The regulatory transition to trivalent seasonal vaccines following B/Yamagata’s disappearance [29], while simplifying current vaccine composition, should not diminish investment in broadly protective IBV vaccine research. The evolutionary trajectory of B/Victoria remains unpredictable, and the lessons learned from B/Yamagata’s extinction emphasize the importance of developing platform-agnostic, rapidly deployable universal vaccine candidates capable of addressing both known and emergent IBV antigenic variants.

## 5. Conclusions

Influenza virus remains a serious global health challenge, highlighting the urgent need for effective universal vaccine development. In parallel with efforts to create combined universal influenza A/B vaccines, IBV-specific vaccines are being actively developed. The importance of the latter lies not only in preventing influenza B but also in the attractive prospect of eradicating IBV from the human population, given the absence of a sustained animal reservoir and the relatively limited phylogenetic divergence of IBV glycoproteins.

Over the last decade, considerable progress has been made in understanding the innate and adaptive immune mechanisms that mediate protection during IBV infection. A key question now is how to optimally induce universal and long-lasting immunity against IBV. Considering the substantial disease burden and socioeconomic impact of IBV infections, addressing these questions to rationally design universal IBV vaccines would provide significant health and societal benefits.

## Figures and Tables

**Table 1:** Commercially available Influenza Vaccines.

Commercial Vaccine	Type	Manufacturer	Age Group	Commentary	Actuality
Fluad Quadrivalent	IIV	Seqirus	≥65 years	Contain adjuvant MF59	2023
Afluria Quadrivalent	IIV	Seqirus	≥6 month		2024
Flucelvax Quadrivalent	IIV	Seqirus	≥6 month		2023
Flulaval Quadrivalent	IIV	GSK	≥6 month	Widely used for massive vaccination	2023
FluMist Quadrivalent	LAIV	AstraZeneca	2–49 years	Intranasal	2023
Fluarix Quadrivalent	IIV	GSK	≥6 month		2023
Fluzone Quadrivalent	IIV	Sanofi Pasteur	≥6 month	Available in high dose version for elder people	2025
Flublok Quadrivalent	rHA	Protein Sciences Corporation/Sanofi Pasteur	≥18 years	Recombinant	2025
Ultrix Qadri	IIV	FORT	≥6 month		2024
Sovigripp	IIV	Microgen	≥6 month	Threevalent, from 18 years version with preservatives	2024
Grippol^®^ plus	IIV	Petrovax Farm	≥6 month	Threevalent, with adjuvant	2025
Flu-M tetra	IIV	FGUP SPbNIIVS FBMA	≥6 month		2025

## Data Availability

No new data were created or analyzed in this study. Data sharing is not applicable to this review.

## Supplementary Materials

The following supporting information can be downloaded at: https://www.mdpi.com/article/10.3390/vaccines14070574/s1, Figure S1: HA aa alignment.
