# Supplementary material for: Influenza B Vaccines: Current Landscape and Novel Development Strategies
Source: Vaccines (Basel). 2026 Jun 29;14(7):574. doi: 10.3390/vaccines14070574 (PMC13418997; doi:10.3390/vaccines14070574)
Supplement: Supplementary file 1 [file vaccines-14-00574-s001.zip › vaccines-4382031-supplementary.pdf]

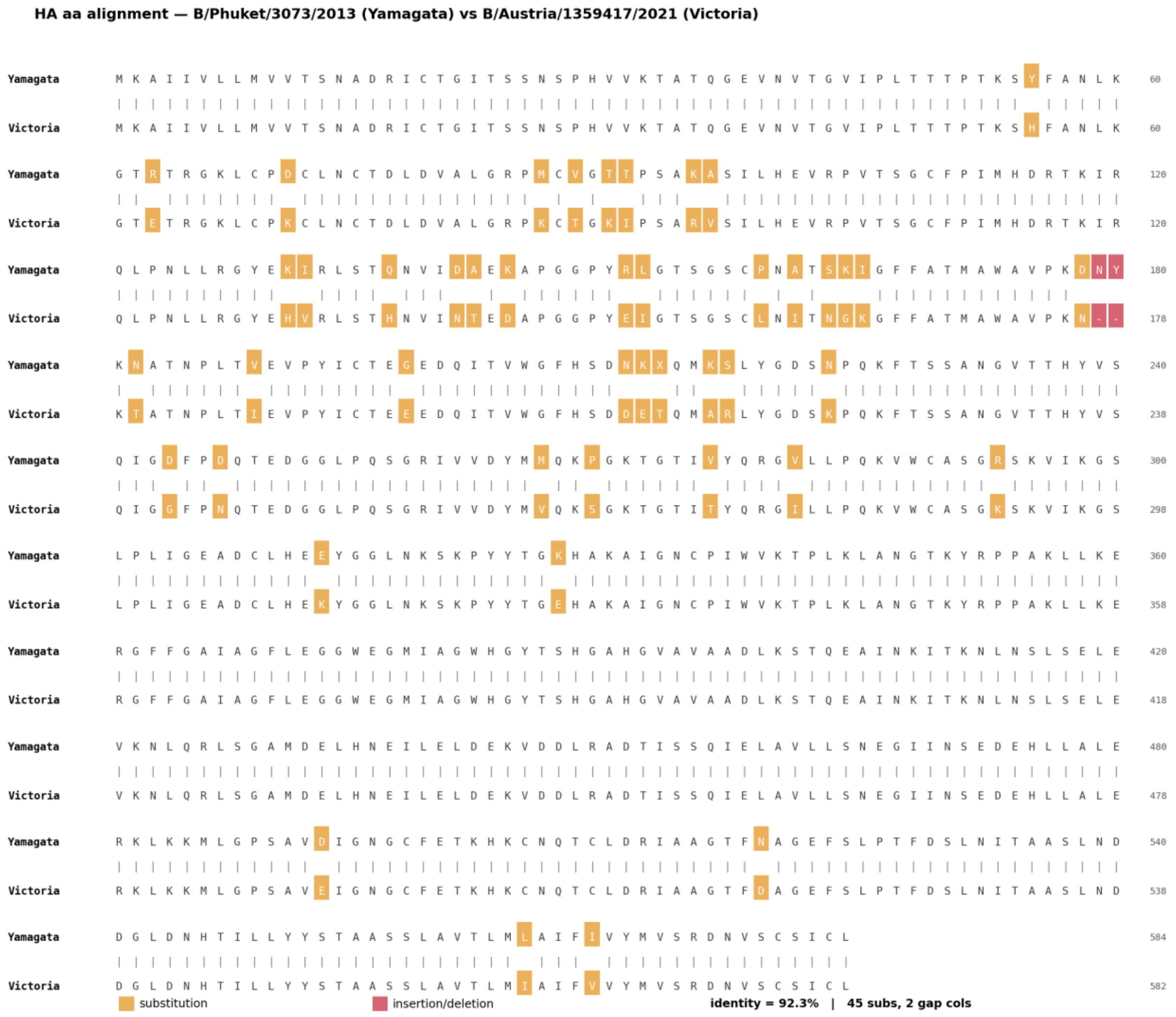

Supplementary Figure S1. Amino-acid sequence alignment of the hemagglutinin (HA) of the contemporary vaccine reference strains of the two influenza B lineages: B/Phuket/3073/2013 (B/Yamagata lineage) and B/Austria/1359417/2021 (B/Victoria lineage). Substituted positions are highlighted in amber and insertions/deletions in red; identical positions are shown in grey. Numbering follows the full-length HA0 precursor. The two proteins share 92.3% amino-acid identity over aligned non-gap columns (45 differing positions, 2 gap columns); sequence variation is concentrated in the HA1 head domain, whereas the HA2 stem is highly conserved.

HA sequences were retrieved from the GISAID EpiFlu database (B/Phuket/3073/2013, EPI_ISL_402395; B/Austria/1359417/2021, EPI_ISL_1519459) and aligned using a global pairwise alignment with the BLOSUM62 substitution matrix (gap-open −10, gap-extend −0.5). One ambiguous residue (X) was present at position 179 of the B/Phuket/3073/2013 sequence (EPI1649072; encoded by an ambiguous RCC codon in the nucleotide record) and was retained as deposited; this position is among the 45 differing columns reported.
